# Potentially preventable hospitalizations and super-utilization of inpatient services among patients with chronic kidney disease in Hawaiʻi

**DOI:** 10.1186/s12882-022-03048-3

**Published:** 2022-12-23

**Authors:** Devashri Prabhudesai, James Davis, John J. Chen, Eunjung Lim

**Affiliations:** Department of Quantitative Health Sciences, John A. Burns School of Medicine, University of Hawaii, Honolulu, HI USA

**Keywords:** Chronic kidney disease, Ambulatory care-sensitive conditions, Super-utilization, Super-utilizers

## Abstract

**Background:**

Chronic kidney disease (CKD) is linked to high morbidity and mortality and increased hospitalization burden. If appropriately managed in the outpatient setting, ambulatory care-sensitive conditions (ACSCs) do not lead to hospitalization. Hospitalizations due to ACSCs are considered as potentially preventable hospitalizations. Patients with recurrent hospitalizations are considered as super-utilizers of inpatient services. The aim of this study is to determine prevalence of potentially preventable hospitalizations and super-utilization of inpatient services among patients with CKD in Hawaiʻi.

**Methods:**

Hawaiʻi statewide inpatient data (2015–2017) were used to identify adult CKD patients with hospitalizations during a 12-month period from the first recorded date of CKD. The associations between the potentially preventable hospitalizations and super-utilization and other key patient demographic and clinical variables (sex, age, ethnicity, insurance type, Charlson comorbidity index (CCI), county of residence, and homelessness indicator) were analyzed using bivariate analysis. Multivariable logistic regression was utilized to assess the associations between the potentially preventable hospitalizations and patient variables.

**Results:**

Approximately 2% of patients reported potentially preventable hospitalizations, and a total of 12.3% patients reported super-utilization. Out of all CKD-specific ACSC hospitalizations, 74.2% were due to heart failure and 25.8% were due to hyperkalemia. Patients who reported super-utilization were more likely to report potentially preventable hospitalization (OR: 5.98, 95%CI: 4.50–7.93) than patients who did not report super-utilization.

**Conclusion:**

This study showed prevalence of potentially preventable hospitalizations and high inpatient utilization among CKD patients in Hawaiʻi. Heart failure and hyperkalemia were the two major causes of CKD-specific ACSC hospitalizations in this cohort. Effective strategies should be employed to improve the outpatient CKD management to reduce hospitalizations and in turn reduce cost.

## Background

Chronic kidney disease (CKD) is one of the most prevalent (10–13%) degenerative diseases with high morbidity and mortality and is considered among the diseases that cause severe public health problems globally [1–3]. More than 10 million cases of CKD were reported in the United States in 2017 [4]. The prevalence of CKD was projected to increase from 14.4% in 2020 to 16.7% in 2030 in adults over 30 years of age [5]. A simulation study estimated that more than 50% of Americans born in 2013 would develop CKD Stage 3+ during their lifetime [6]. A 10-year population health study showed that Filipinos and Native Hawaiians have a higher risk of early kidney damage [7]. Clinical progression of CKD can lead to end stage kidney disease (ESKD), dependence on dialysis, development of cardiovascular complications, and/or exponentially increased absolute risk for death with declining kidney function [8].

Ambulatory care-sensitive conditions (ACSCs) are health conditions that can be managed by good outpatient care and hence potentially prevent hospitalization related to ACSCs. ACSCs are globally recognized as a measure of competence of ambulatory and primary healthcare services [9]. In 2020, Sentell et al. reported the estimated cost of ACSC hospitalizations for the state of Hawaiʻi at over $250 million [10]. In CKD patients, potentially preventable hospitalization is deemed as inpatient service utilization for a CKD-specific ACSC. Hyperkalemia, malignant hypertension, heart failure, and volume overload are the four CKD-specific ACSCs [9]. CKD management (treatment of albuminuria, reduction of cardiovascular risk, avoiding exposure to potential nephrotoxins, drug dosing adjustments, etc.), if inadequate, often leads to increased hospitalization burden [11, 12] which is one of the highly expensive types of healthcare utilization [13]. Increased hospitalization rates have been reported among people with high serum creatinine levels [14], and high hospitalization burden is reported in persons with Stage 3 and Stage 4 non-dialysis-dependent CKD [15]. In 2016, Ronksley et al. showed that during a median follow-up of 3 years, approximately one in four CKD patients was hospitalized for a CKD-specific ACSC [16]. Another study showed that during a mean follow-up of 2.4 years, 5.8% of emergency department encounters were for CKD-related ACSCs. Approximately one third of the encounters resulted in hospitalization [17].

Patients who disproportionately use high inpatient or emergency room services are termed as super-utilizers and they pose a significant burden on the healthcare system [18]; disproportionate healthcare utilization can be defined as recurrent hospitalization or a small proportion of population accounting for substantial healthcare spending. A study conducted to identify patients at high risk of super-utilization of inpatient and emergency services found diagnosis of CKD as one of the strongest predictors for super-utilization [19]. Super-utilizers often suffer from multiple chronic conditions, and an estimated 0 to 50% of total spending is accounted to super-utilizers who make up 3 to 5% of the US population [20].

Given the evidence of burden of super-utilization of inpatient services among CKD patients, it is important to determine the prevalence of potentially preventable hospitalizations and identify super-utilization among CKD patients. To our knowledge prevalence of CKD-specific ACSC related hospitalization and super-utilization among patients with CKD in Hawaiʻi have not been explored before. To fill this gap, we estimate the prevalence of CKD-specific ACSC related hospitalizations and super-utilization among hospitalized CKD patients in Hawaiʻi and examine association between CKD-specific ACSC related hospitalizations and super-utilization adjusting for demographic and clinical characteristics of those patients with CKD-specific ACSC related hospitalizations.

## Methods

### Data source

We analyzed the deidentified statewide inpatient data from Hawaiʻi from 2015 to 2017. Data from all 23 acute care hospitals in Hawaiʻi were included.

### Sample

The study included adults (18 years and older) with hospitalizations during the follow-up period of 12 months from the index hospitalization that occurred between January 2015 and August 2016; index hospitalization was defined as the first hospitalization when CKD was reported. Presence of CKD was determined by using International Statistical Classification of Diseases and Health Related Problems, Ninth (ICD-9-CM) and Tenth (ICD-10-CM) Revisions Clinical Modifications [21, 22]. For CKD, ICD-9-CM codes: 585, 585.1, 585.2, 585.3, 585.4, 585.5, 585.6, 585.9 and ICD-10-CM codes: N18.x were used.

**Fig. 1:**
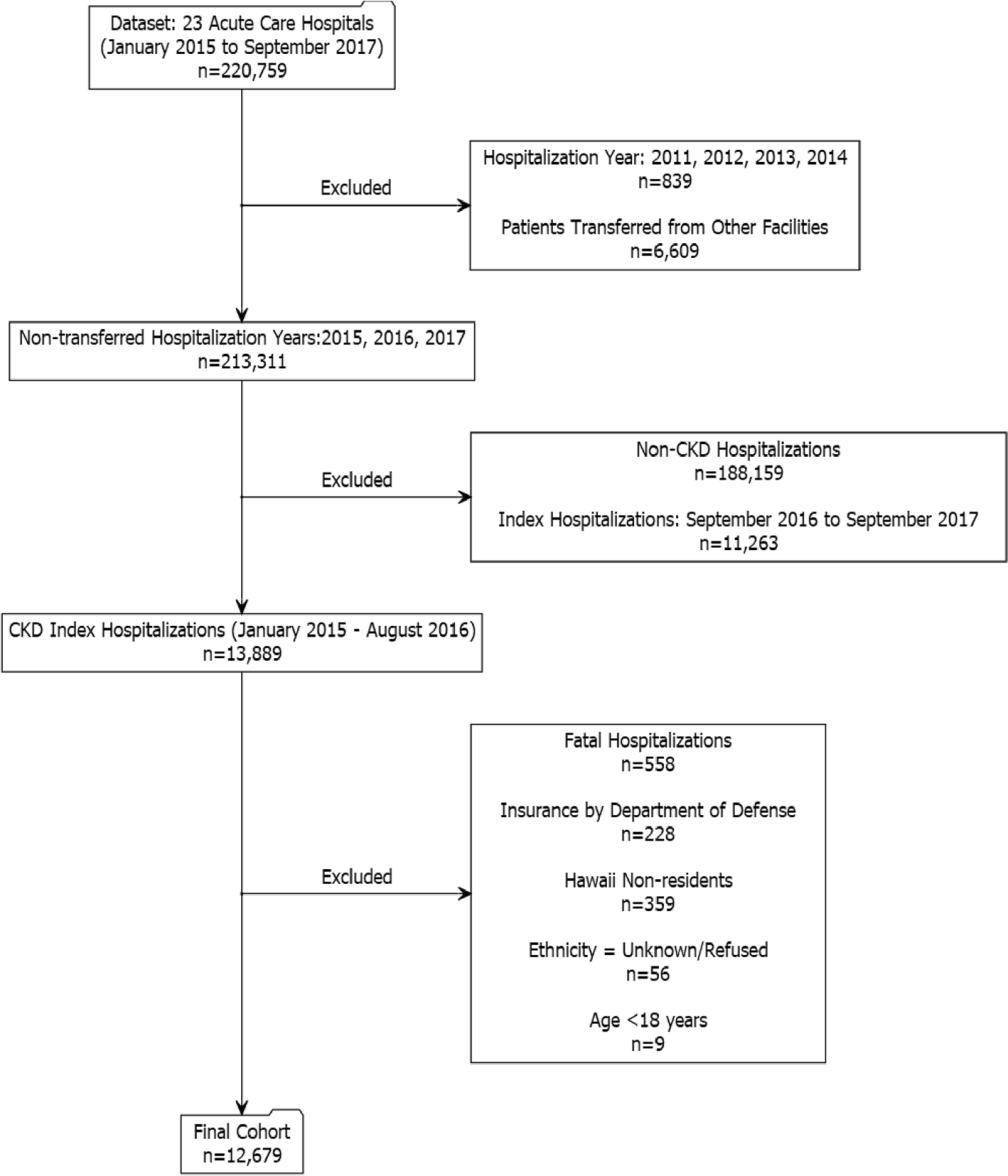
Flowchart of the process determining the study cohort

Patients who died during the index hospitalization or during the follow-up period of 12 months from the index hospitalization were excluded from the analysis. Since payer (insurance) category ‘Department of Defense (DoD)’ did not report race/ethnicity data consistently, patients with payer DoD were excluded from the analysis. Also, patients were excluded if they did not report race/ethnicity or reported it as ‘unknown’ or if patients were not a resident of Hawaiʻi. A cohort of 12,679 CKD patients was analyzed. Detailed process of determining the study cohort is illustrated in Fig. 1. This study was deemed exempt from the review by institutional review board (2017–00821).

### Measures

#### Dependent variable

*Potentially Preventable Hospitalizations among CKD Patients*: An ACSC is a health condition that can be managed by good outpatient care and can potentially prevent subsequent hospitalization. In CKD patients, potentially preventable hospitalization is deemed as inpatient service utilization for a CKD-specific ACSC. The four causes of CKD-specific ACSCs were hyperkalemia, malignant hypertension, volume overload, and heart failure [9, 16, 17]. Table 1 contains the ICD-9-CM and ICD-10-CM codes used to identify the CKD-specific ACSCs. The ACSC hospitalization within 12 months of follow-up period was dichotomized as “Reported” (at least one ACSC related hospitalization) or “Not reported” (no ACSC related hospitalizations).

**Table 1:** ICD-9-CM and ICD-10-CM codes used to define CKD-specific ACSCs [9]

ACSC	ICD-9-CM	ICD-10-CM
Hyperkalemia	276.7	E87.5
Malignant hypertension	401.0, 402.00, 402.01, 403.0, 404.0, 405.0	I10.1
Volume overload	276.6	E87.7
Heart failure	428.x, 398.91, 402.01, 402.11, 402.91, 404.01, 404.03, 404.11, 404.13, 404.91, 404.93, 425.4–425.9	I09.9, I11.0, I13.0, I13.2, I25.5, I42.0, I42.5-I42.9, I43.x, I50.x

IC﻿D-9-CM: International Statistical Classification of Diseases, ninth revision, Clinical modification; ICD-10-CM: International Statistical Classification of Diseases, tenth revision, Clinical modification; CKD: Chronic Kidney Disease; ACSCs: Ambulatory Care Sensitive Conditions

Independent variable: *Super-utilization*: We measured super-utilization as 3 or more hospitalizations after the index hospitalization, within 12 months of follow-up [20]. The super-utilization variable was dichotomized as “Present” or “Absent”.

Covariates: Sex, age, ethnicity, insurance type, Charlson comorbidity index (CCI), county [17–19], and homelessness indicator [23].

*Age group*: Age was categorized as “Less than 65 years” and “65 years and over”.

*Ethnicity*: Race/Ethnicity was categorized as “White”, “Chinese”, “Filipino”, “Japanese”, “Native/Part Native Hawaiian”, “Pacific/Other Pacific Islander”, and “Other”.

*Insurance type*: Patient’s insurance type was categorized as “Medicaid”, “Medicare”, “Private insurance”, and “Other” (including self-pay and miscellaneous types).

*CCI*: CCI is a weighted sum of the 16 comorbid conditions (CKD was excluded) derived from ICD-9-CM and ICD-10-CM coding algorithm. The CCI was categorized as “CCI 0”, “CCI 1”, “CCI 2”, “CCI 3 or above”.

*County*: County of patients’ residence was categorized as “Oahu County” vs. “Other Counties” (includes Hawaii County, Kauai County, Maui County).

*Homelessness indicator*: Homelessness indicator was dichotomized as “Yes” vs. “No”.

### Statistical analysis

Descriptive statistics was generated with means and standard deviations for the continuous variables and frequencies and percentages for the categorical variables. Bivariate analyses were conducted to evaluate the associations between the ACSC hospitalizations and other variables using Pearson’s Chi-squared tests and Fisher’s exact tests for the categorical variables. Multivariable logistic regression was used to assess the associations between the ACSC Hospitalizations and independent variable, adjusting for covariates. Adjusted odds ratios (ORs) and their 95% confidence intervals (CIs) were estimated. Multicollinearity was assessed by determining the variance inflation factor (VIF), with a VIF value less than 10 being regarded as acceptable, and c-statistic was calculated to summarize the overall predictive accuracy of the model. Analyses were performed in R version 4.0.2, and the significance level was 0.05.

## Results

Out of the total of 12,679 patients, approximately 2% reported at least one ACSC hospitalization within the 12-month follow-up period and 12.3% reported super-utilization. In this cohort, 56.4% were males and a majority belonged to the “65 years and over” age group (69.7%), were Japanese (20.9%), had Medicare insurance (73.6%), lived in Oahu County (76.4%), and were not homeless (98.3%). The CCI scores “1”, “2”, and “3 or above” were similar (29.3, 29.5, and 30.3%, respectively). Super-utilization, Age group, Insurance type, CCI, and Homelessness indicator were significantly associated with ACSC hospitalizations (Table 2).

**Table 2:** Patient Characteristics by ACSC Hospitalization

		At least 1 ACSC hospitalization		
Variables	Total n (%) 12,679	Not Reported n (%) 12,466 (98.3%)	Reported n (%) 213 (1.7%)	*p*
Super-utilization				< 0.001
Absent	11,116 (87.7%)	11,007 (88.3%)	109 (51.2%)	
Present	1563 (12.3%)	1459 (11.7%)	104 (48.8%)	
Sex				0.13
Female	5532 (43.6%)	5450 (43.7%)	82 (38.5%)	
Male	7147 (56.4%)	7016 (56.3%)	131 (61.5%)	
Age group				< 0.001
65 years and over	8831 (69.7%)	8720 (70.0%)	111 (52.1%)	
Less than 65 Years	3848 (30.3%)	3746 (30.0%)	102 (47.9%)	
Ethnicity				0.07
White	2211 (17.4%)	2181 (17.5%)	30 (14.1%)	
Chinese	778 (6.1%)	768 (6.2%)	10 (4.7%)	
Filipino	2469 (19.5%)	2432 (19.5%)	37 (17.4%)	
Japanese	2648 (20.9%)	2612 (21.0%)	36 (16.9%)	
Native/Part Native Hawaiian	2349 (18.5%)	2296 (18.4%)	53 (24.9%)	
Pacific/Other Pacific Islander	1086 (8.6%)	1064 (8.5%)	22 (10.3%)	
Other	1138 (9.0%)	1113 (8.9%)	25 (11.7%)	
Insurance type				< 0.001
Medicaid	1353 (10.7%)	1305 (10.5%)	48 (22.5%)	
Medicare	9332 (73.6%)	9194 (73.8%)	138 (64.8%)	
Private Insurance	1880 (14.8%)	1857 (14.9%)	23 (10.8%)	
Other	114 (0.9%)	110 (0.9%)	4 (1.9%)	
CCI score				< 0.001
CCI score 0	1382 (10.9%)	1372 (11.0%)	10 (4.7%)	
CCI score 1	3721 (29.3%)	3685 (29.6%)	36 (16.9%)	
CCI score 2	3738 (29.5%)	3661 (29.4%)	77 (36.2%)	
CCI score 3 or above	3838 (30.3%)	3748 (30.1%)	90 (42.3%)	
County				0.7
Oahu	9687 (76.4%)	9522 (76.4%)	165 (77.5%)	
Other Counties	2992 (23.6%)	2944 (23.6%)	48 (22.5%)	
Homelessness indicator				0.01
No	12,461 (98.3%)	12,257 (98.3%)	204 (95.8%)	
Yes	218 (1.7%)	209 (1.7%)	9 (4.2%)	

*ACSC* Ambulatory Care Sensitive Condition; *CCI* Charlson Comorbidity Index;* n* Number of patients; *%* Percentage

In this cohort, 25.8% ACSC hospitalizations were due to hyperkalemia and 74.2% were due to heart failure; no ACSC hospitalizations were due to malignant hypertension or volume overload. Out of patients who reported at least one ACSC hospitalization during the 12-month follow-up period, majority were male (61.5%), 65 years or over (52.1%), Native/Part Native Hawaiian (24.9%), had Medicare insurance (64.8%), lived on Oahu (77.5%), were not homeless (95.8%), and reported CCI 3 or above (42.3%) (Table 2).

**Fig. 2:**
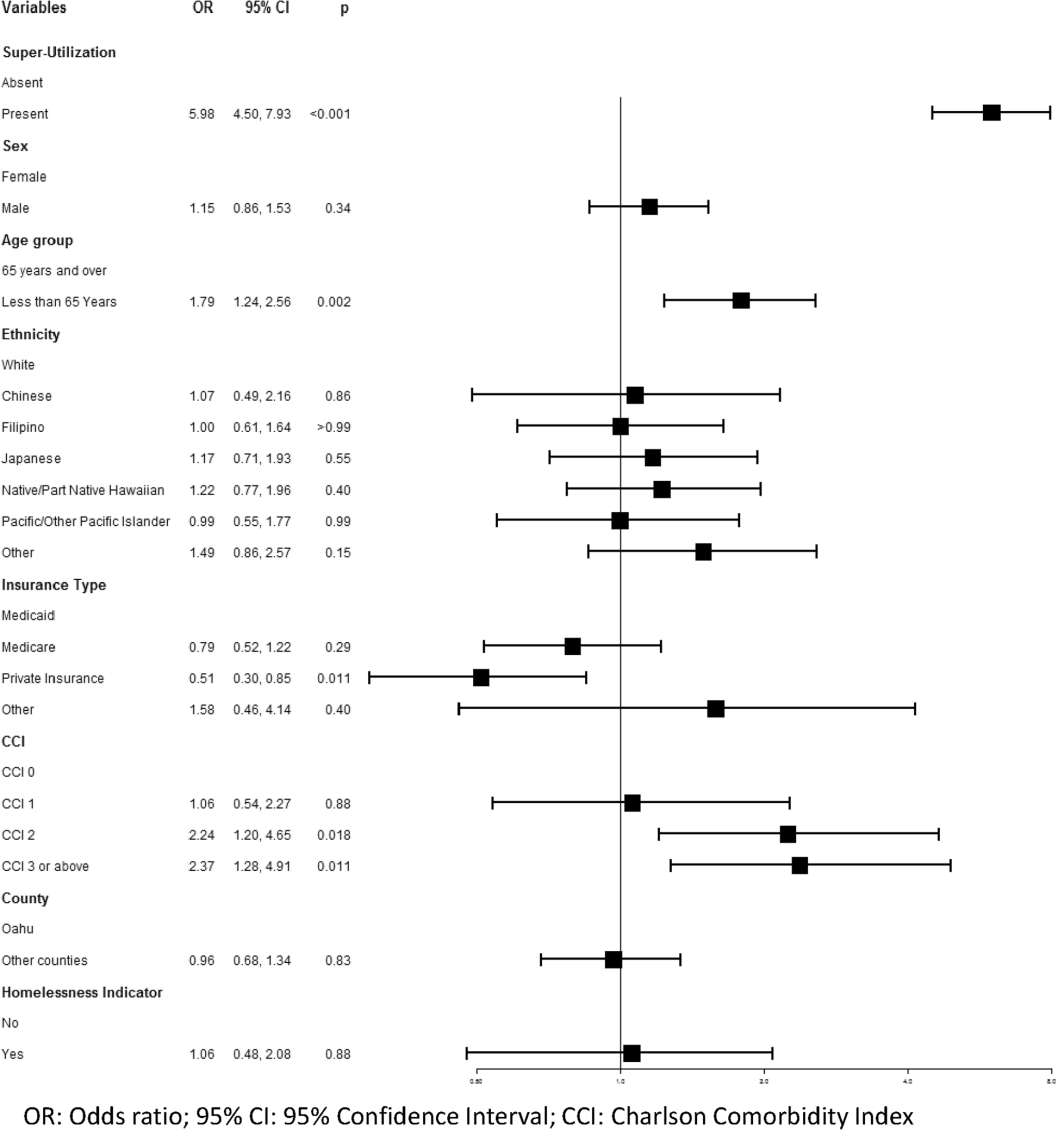
Adjusted multivariable logistic regression model shows super-utilization is significantly associated with ACSC hospitalization (*p* < 0.001)

Figure 2 shows the results from the multivariable logistic regression analysis. The adjusted model indicated that super-utilization was significantly associated with the ACSC hospitalizations (*p* < 0.001). Compared to non-super-utilization, patients who reported super-utilization were more likely to report potentially preventable hospitalization (OR: 5.98, 95%CI: 4.50–7.93). Patients younger than 65 years of age were more likely to report potentially preventable hospitalization compared to patients aged 65 years or older (OR: 1.79, 95%CI: 1.24–2.56). Patients with private insurance were less likely to report potentially preventable hospitalization than patients with Medicaid insurance (OR: 0.51; 95%CI: 0.30–0.85). Patients with ‘CCI of 2’ (OR: 2.24; 95%CI: 1.20–4.65) and ‘CCI of 3 or above’ (OR: 2.37; 95%CI: 1.28–4.91) were more likely to report hospitalization due to ACSC compared to patients with ‘CCI of 0’. No significant association was found between ACSC hospitalization and sex, ethnicity, county of residence, and homelessness indicator. The c-statistic of this multivariable logistic regression model was 0.77 and no multicollinearity was observed between the variables.

## Discussion

In 2021, 15% of US adults were estimated to have CKD [24]. In this study on average, patients were found to have CKD in approximately 15% of hospitalizations. Our study examined prevalence of potentially preventable hospitalizations (CKD-specific ACSC hospitalizations) and super-utilization of inpatient care among CKD patients in Hawaiʻi. Many studies mention that CKD patients, when compared to the patients without CKD, are more likely to be hospitalized and have a high risk of hospital-acquired complications and readmission [11, 14, 15, 25]; however, to our knowledge only a few have explored potentially preventable hospitalizations and high-inpatient use among CKD patients [9, 16, 17]. We found that among the study cohort of patients with CKD, approximately 2% patients reported hospitalizations due to ACSCs during a 12-month follow-up period, which was lower than the ACSC related hospitalizations among CKD patients in Canada (10.9%) with a median follow-up of 4.1 years [9]. The difference in the prevalence could be attributed to the different follow-up periods between the studies. Our study also showed that 74.2% of the ACSC hospitalizations were due to heart failure and remaining 25.8% were due to hyperkalemia. Similar results were seen from another study that investigated emergency department (ED) use due to CKD-related ACSCs and found that heart failure constituted 80.0% of all potentially preventable ED encounters [17]. A study conducted in the US among veterans with a history of CKD, showed an association between iron deficiency and higher hospitalization risk due to heart failure [26]. Further studies are warranted to evaluate effects of iron deficiency among CKD patients in Hawaiʻi.

In the current study we explored super-utilization of inpatient care among Hawaiʻi CKD patients and its effect on potentially preventable hospitalizations. Although 1.7% of the study cohort reported ACSC hospitalization, almost half of them (48.8%) reported super-utilization, and our study found significant association between super-utilization and CKD specific potentially preventable hospitalization. A study conducted in Utah also showed that CKD was significantly associated with persistent super utilizer (≥3 hospitalizations per year for 3 years) [27]. Another study conducted in Massachusetts among non-elderly adults (Age: 18 to 64 years) found super-utilizers (defined as high-cost patients) are more likely to attract higher levels of preventable spendings (in terms of preventable hospitalization or preventable ED visits) compared to non-super-utilizers (non-high-cost patients) [28]. We found that CKD patients aged less than 65 years are significantly more likely to report potentially preventable hospitalizations than elderly patients in Hawaiʻi. Also, a Canadian study showed that persistent high utilizers of the inpatient services were usually younger (often in 18 to 44 years age group) compared to the episodic and non-high utilizers [16]. Future studies are warranted to explore factors contributing to potentially preventable hospitalizations among non-elderly patients with CKD and their super-utilization status.

The current study shows that compared to Medicaid, CKD patients with private insurance are significantly less likely to report potentially preventable hospitalization. The rate of potentially preventable hospitalizations among older patients with Medicaid was higher than the rate in patients without Medicaid [29]. The Massachusetts study showed that substantial preventable spending was incurred by the non-elderly super-utilizers with Medicaid [28]. A study examining associations between multimorbidity and hospitalization rates among CKD patients showed that rate of hospitalization is two to three times higher in CKD patients with multimorbidity [25]. Previous studies have shown that comorbid conditions are also significantly associated with potentially preventable hospitalizations among CKD patients [9, 16]. Our findings extend the association that the ‘CCI of 2’ and ‘CCI of 3 or above’ are significantly associated with potentially preventable hospitalizations and CKD patients with either of these CCI scores have more than two times higher odds of being hospitalized due to the ACSCs. In our study, congestive heart failure (37.0%), chronic pulmonary disease (28.3%), diabetes without complications (27.6%), diabetes with complications (27.9%), and myocardial infarction (19.0%) were the common comorbid conditions. A previous study has shown that ACSCs of heart failure, hyperkalemia, and volume overload were strongly associated with comorbidities such as diabetes, chronic liver disease, and heart failure [17]. The associations between comorbid conditions and super-utilization can be explored in future studies.

One of the limitations of this study is that all patients with fatal hospitalizations during the defined 12-month follow-up period were excluded from the analysis. Another limitation is that since CKD severity cannot be accurately defined using ICD-9 or ICD-10 codes alone and the possibility of misclassification of ICD-9 or ICD-10 codes or errors in data entry, CKD severity was not included as one of the covariates. Also, Hawaiʻi inpatient data were administrative data and lack covariates such as medications used, education, family income, and access to primary care for a more informative analysis. Studies have shown that better access to primary care or high attachment to physician decreases the chances of potentially preventable hospitalizations [9, 16, 29]. Further studies are required to explore the association between access to primary care and potentially preventable hospitalizations among CKD patients. Also, while this secondary data analysis focuses on the accessible data from 2015 to 2017, it is plausible that some of the conclusions might change as current data become available for analysis. It is possible that some patients with CKD or some ACSC hospitalizations could have been missed due to misclassification of ICD-9 or ICD-10 codes or errors in data entry. Lastly, as laboratory values were not used to define CKD, some patients with CKD could have been missed.

The strength of this study is that patient level multiple hospitalizations can be tracked across various hospitals and within the same hospital using a unique patient identifier. This helps providing a true patient level information. Also, that Hawaiʻi inpatient data includes data on all hospitalizations and allows for reliable analysis. This study adds to the very limited literature on the prevalence of potentially preventable hospitalizations among CKD patients and super-utilization of the inpatient services by the CKD patients in Hawaiʻi.

As similarities were found between our study and the Canadian studies [9, 16, 17], we expect that our findings can be extended to other developed countries with similar healthcare systems. However, due to the diversity of the Hawaiian population, there is a possibility that our study may not be directly applicable to other populations with homogenic racial/ethnic distribution. Further studies are needed to investigate the generalizability of our findings to other U.S. states or countries. Given the significant association between super-utilization and CKD specific potentially preventable hospitalization, as found in our study, systematic identification and management of signs/symptoms leading to heart failure and hyperkalemia in the outpatient settings as well as the early capture or identification of these potential super-utilizers would be the key for targeted interventions to improve health and reduce burden.

## Conclusion

During the 12-month follow-up period of the patients with CKD, approximately 2.0% reported potentially preventable CKD-specific ACSC hospitalizations and almost half of those were super-utilizers of inpatient care. The ACSC hospitalizations were significantly affected by age, super-utilization, insurance type, and CCI. In this cohort the majority of CKD-specific ACSC hospitalizations were due to heart failure followed by hyperkalemia. Identifying the causes of ACSC hospitalizations will help formulating policies for effective and focused outpatient management for patients with CKD and in turn reduce the risk of heart failure and hyperkalemia and ultimately reduce the cost associated with preventable hospitalizations.

## Data Availability

The datasets used and/or analyzed during the current study are not publicly available because the company, Hawaii Health Information Corporation (HHIC), that had been collecting this statewide data closed its business in 2017 but are available from the corresponding author on reasonable request.

## Abbreviations

CKD: Chronic kidney disease
ESKD: End stage kidney disease
ACSCs: Ambulatory care-sensitive conditions
ICD-9-CM: International Statistical Classification of Diseases and Health Related Problems, Ninth Revision Clinical Modifications
ICD-10-CM: International Statistical Classification of Diseases and Health Related Problems, Tenth Revision Clinical Modifications
DoD: Department of Defense
CCI: Charlson Comorbidity Index
ORs: Odds ratios
CIs: Confidence intervals
VIF: Variance inflation factor

## References

[1] Genovese C, Belvis AGDE, Rinaldi M, Manno V, Squeri R, Fauci LA, Tabbi P (2018). Quality and management care improvement of patients with chronic kidney disease: from data analysis to the definition of a targeted clinical pathway in an Italian Region. J Prev Med Hyg.

[2] Ammirati AL (2020). Chronic kidney disease. Rev Assoc Med Bras(1992).

[3] Arcoraci V, Barbieri MA, Rottura M (2021). REPOSI investigators. Kidney disease management in the hospital setting: a focus on inappropriate drug prescriptions in older patients Front Pharmacol.

[4] GBD chronic kidney disease collaboration (2020). Global, regional, and national burden of chronic kidney disease, 1990-2017: a systematic analysis for the global burden of disease study 2017. Lancet..

[5] Hoerger TJ, Simpson SA, Yarnoff BO (2015). The future burden of CKD in the United States: a simulation model for the CDC CKD initiative. Am J Kidney Dis.

[6] Grams ME, Chow EKH, Segev DL, Coresh J (2013). Lifetime incidence of CKD stages 3-5 in the United States. Am J Kidney Dis.

[7] Kataoka-Yahiro MR, Davis J, Rhee CM, Wong L, Hayashida G (2020). Racial/ethnic differences in early detection and screening for chronic kidney disease among adults in Hawaii: a 10-year population health study. Prev Chronic Dis.

[8] Hansrivijit P, Chen YJ, Lnu K (2021). Prediction of mortality among patients with chronic kidney disease: a systematic review. World J Nephrol.

[9] Wiebe N, Klarenbach SW, Allan GM, Alberta Kidney Disease Network (2014). Potentially preventable hospitalization as a complication of CKD: a cohort study. Am J Kidney Dis.

[10] Sentell TL, Seto TB, Quensell ML, Malabed JM, Guo M, Vawer MD, Braun KL, Taira DA (2020). Insights in public health: outpatient care gaps for patients hospitalized with ambulatory care sensitive conditions in Hawai'i: beyond access and continuity of care. Hawaii J Health Soc Welf.

[11] Srivastava A, Cai X, Mehta R (2021). CRIC study investigators. Hospitalization trajectories and risks of ESKD and death in individuals with CKD. Kidney Int Rep.

[12] Chen TK, Knicely DH, Grams ME (2019). Chronic kidney disease diagnosis and management: a review. JAMA..

[13] Liang L, Moore B, Soni A (2020). National inpatient hospital costs: the most expensive conditions by payer, 2017: statistical brief #261. Healthcare cost and utilization project (HCUP) statistical briefs [internet].

[14] Go AS, Chertow GM, Fan D, McCulloch CE, Hsu CY (2004). Chronic kidney disease and the risks of death, cardiovascular events, and hospitalization. N Engl J Med.

[15] Triozzi JL, Niu J, Walther CP, Winkelmayer WC, Navaneethan SD (2020). Hospitalization and critical illness in chronic kidney disease. Cardiorenal Med.

[16] Ronksley PE, Hemmelgarn BR, Manns BJ (2016). Potentially preventable hospitalization among patients with CKD and high inpatient use. Clin J Am Soc Nephrol.

[17] Ronksley PE, Tonelli M, Manns BJ (2017). Emergency department use among patients with CKD: a population-based analysis. Clin J Am Soc Nephrol.

[18] Rinehart DJ, Oronce C, Durfee MJ (2018). Identifying subgroups of adult superutilizers in an urban safety-net system using latent class analysis: implications for clinical practice. Med Care.

[19] Ziring J, Gogia S, Newton-Dame R, Singer J, Chokshi DA (2018). An all-payer risk model for super-utilization in a large safety net system. J Gen Intern Med.

[20] Surbhi S, Graetz I, Wan JY, Gatwood J, Bailey JE (2020). Medication nonadherence, mental health, opioid use, and inpatient and emergency department use in super-utilizers. Am J Manag Care.

[21] Jalal J, Anand EJ, Venuto R, Eberle J, Arora P (2019). Can billing codes accurately identify rapidly progressing stage 3 and stage 4 chronic kidney disease patients: a diagnostic test study. BMC Nephrol.

[22] Shih YJ, Kuo YT, Ho CH, Wu CC, Ko CC (2019). Incidence and risk of dialysis therapy within 30 days after contrast enhanced computed tomography in patients coded with chronic kidney disease: a nation-wide, population-based study. PeerJ..

[23] Crews DC, Novick TK (2019). Social determinants of CKD hotspots. Semin Nephrol.

[24] Centers for Disease Control and Prevention. Chronic Kidney Disease in the United States, 2021. 2021. https://www.cdc.gov/kidneydisease/publications-resources/ckd-national-facts.html. Accessed 19 Nov 2022.

[25] Sullivan MK, Jani BD, McConnachie A (2021). Hospitalisation events in people with chronic kidney disease as a component of multimorbidity: parallel cohort studies in research and routine care settings. BMC Med.

[26] Cho ME, Hansen JL, Sauer BC, Cheung AK, Agarwal A, Greene T (2021). Heart failure hospitalization risk associated with iron status in veterans with CKD. Clin J Am Soc Nephrol.

[27] Luo J, Collier W, Magno-Padron D (2022). Characteristics of nonelderly adult health care persistent super utilizers in Utah. Popul Health Manag.

[28] Figueroa JF, Frakt AB, Lyon ZM, Zhou X, Jha AK (2017). Characteristics and spending patterns of high cost, non-elderly adults in Massachusetts. Healthc (Amst).

[29] Mahmoudi E, Kamdar N, Furgal A, Sen A, Zazove P, Bynum J (2020). Potentially preventable hospitalizations among older adults: 2010-2014. Ann Fam Med.

